# Gingival Melanin Depigmentation by 808 nm Diode Laser: Report of a Case

**DOI:** 10.1155/2020/8853086

**Published:** 2020-07-08

**Authors:** Jade Chagra, Adel Bouguezzi, Sameh Sioud, Hajer Hentati, Jamil Selmi

**Affiliations:** ^1^Department of Medicine and Oral Surgery, University Dental Clinic of Monastir, Tunisia; ^2^University of Monastir, Faculty of Dental Medicine, Oral Health and Oro-Facial Rehabilitation Laboratory Research (LR12ES11), 5019 Monastir, Tunisia

## Abstract

Gingival hyperpigmentation frequently poses an aesthetic problem, especially in patients with gingival smile. This paper presents the use of a 808 nm pulsed diode laser for gingival depigmentation in a 22-year-old male patient, with a frequency of 20,000 Hz, a peak power of 5 W, and a pulse width of 26 microseconds, using a 400 nm flexible optic fiber. The hyperpigmented gingival tissue was removed without bleeding or postoperative pain. Three weeks later, the gum resumed its normal, firm, and pink appearance. No significant recurrence was noted after a follow-up of 4 months. However, perfect control of this device is necessary to avoid certain consequences such as bone exposure or gingival fenestrations.

## 1. Introduction

Nowadays, expectations in terms of aesthetics have become more important. That is why gum pigmentation, especially in areas uncovered by the smile, has become a frequent reason of consultation [1]. Melanin, melanoid, oxyhemoglobin, reduced hemoglobin, and carotene are pigments involved in gum pigmentation. Gingival hyperpigmentation generally results in the overproduction of an endogenous pigment which is melanin [2]. This type of excessive pigmentation presents as a diffuse brown and light brown spots with irregular contours [1]. The accumulation of melanin can be physiological and called “racial pigmentation” or caused by several endogenous and exogenous factors, including tobacco [3].

Hyperpigmentation has no gender predilection and may be observed in all races and at all ages. Given the considerable interest in gingival depigmentation, several treatments have been developed including scalpel gingivectomy, chemical treatment, cryosurgery, electrosurgery, abrasion with a diamond bur, and laser surgery [3]. These last years, laser dentistry has replaced many traditional dental procedures, making treatments more precise and less painful.

The purpose of this work is to explain the therapeutic approach undertaken in a 22-year-old patient who underwent gingival depigmentation treatment with a diode laser.

## 2. Clinical Case

A 22-year-old nonsmoking patient of brown race was referred to the department of oral medicine and surgery at the University Dental Clinic of Monastir, with a chief complaint of a “dark-color upper gum.” He reported noticing darkening of the gums 10 years ago. The lesion was asymptomatic and has remained stable since then. He had no significant personal history and denied taking any medication or other substances that may have caused this pigmentation.

**Figure 1:**
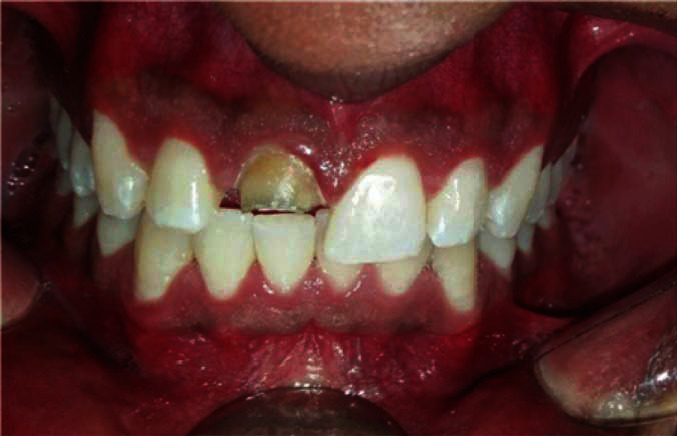
Initial clinical appearance with melanic pigmentations.

In clinical examination, periodontal tissues were healthy, but bilateral melanin pigmentation was present, associated with a fractured upper central incisor. A pigmentation index as mentioned by Kumar et al. [4] was used to determine the level of pigmentation, and the score was diagnosed as “3” (diffuse brown to black pigmentation, marginal, and attached) (Figure 1).

At first sight, the diagnoses evoked by this oral pigmentation of melanin were gingival pigmentation of exogenous origin (drugs, tobacco, metals,…), racial pigmentation, and Addison's disease. However, given the diffuse, bilateral appearance of these pigmentations which are not of recent appearance, the absence of smoking history or drug use, the patient's race, and the absence of systemic involvement, a final diagnosis of physiological gingival pigmentation (ethnic/racial) was established.

Following the patient's request, a laser depigmentation procedure was planned after obtaining the patient's consent.

A semiconductor diode surgical laser unit (Elexxion pico 808 nm diode laser, Elexxion AG, Singen, Germany) was used for the procedure; it is a digitally pulsed diode laser with a frequency of 20,000 Hz, a peak power of 5 W, and a pulse width of 26 microseconds. With this setting, the laser beam is “on” the tissue for only 26 microseconds which makes around one-third of the total cycle. The rest of the time is to allow the tissue to cool down. The semiconductor diode laser was operated in a contact method using a 400 nm flexible fiber optic handpiece.

Special eyeglasses were worn by the patient and the staff to fulfill the Food and Drug Administration laser safety rules. After a local infiltration of anesthesia, the properly initiated tip of the diode laser was angled at an external bevel of 45 degrees. The melanin-pigmented gum was resected by circular movement of the fiber with a slight contact on the tissue, avoiding teeth structures.

**Figure 2:**
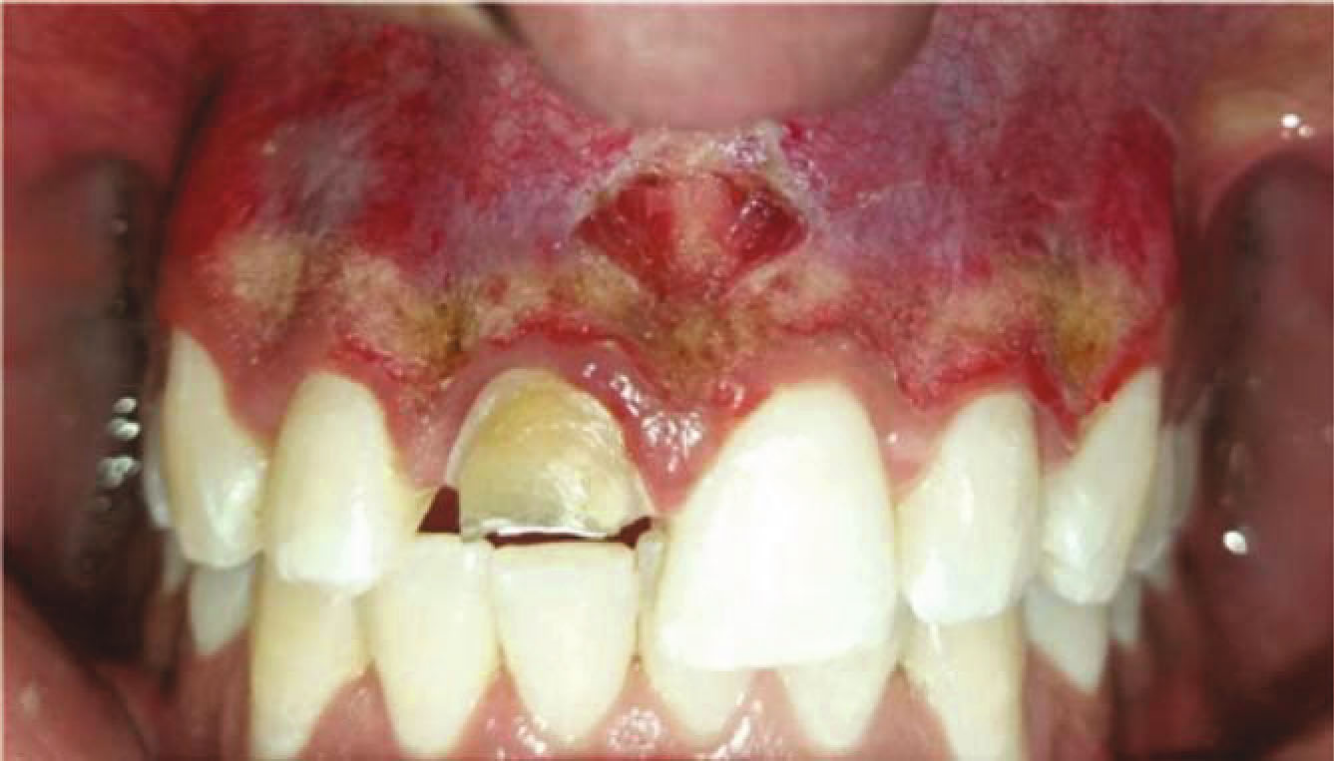
Clinical appearance immediately after the diode laser procedure and labial frenectomy.

This technique was performed on the entire pigmented anterosuperior attached gingiva for the deepithelialization procedure. Saline-soaked gauze was used to remove epithelial remnant. This procedure was repeated until the desired depth of tissue removal was reached, combined with a labial frenectomy (Figure 2). The treatment time was two minutes compared to the ablative technique time up to 30 minutes in an area of first molar to first molar.

Analgesics and 0.2% chlorhexidine mouthwash were prescribed.

**Figure 3:**
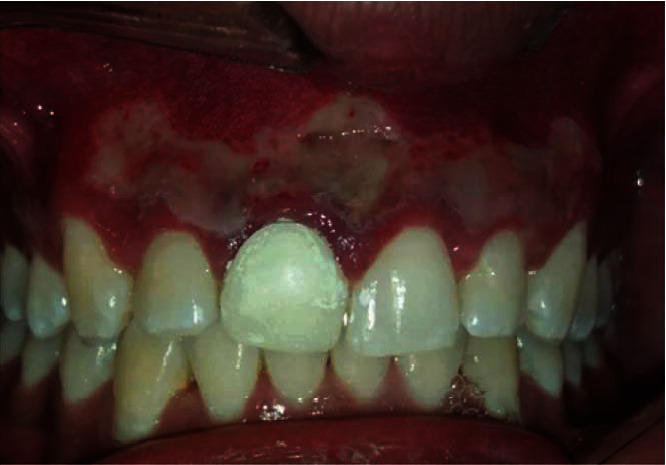
Clinical image showing a fibrin layer that completely covers the “laser wound” 6 hours after the operation.

**Figure 4:**
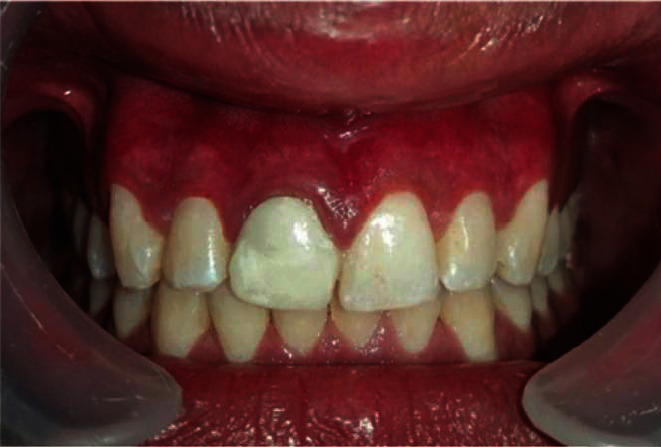
Clinical image of the upper anterior gingiva 4 months after, with no recurrence.

The patient did not report any postoperative pain, swelling, or other complications at the first and subsequent visits. It should be noted that a greyish-white fibrin layer completely covers the “laser wound” 6 hours after the operation (Figure 3), which should not be misinterpreted as secondary infection. Four months later, the patient had no symptoms or signs of gingival melanin pigmentation (Figure 4).

## 3. Discussion

Various methods are used for gum depigmentation with varying degrees of success and recurrence rates. Today, the laser has largely found its place among the therapeutic arsenal used in depigmentation. Several lasers have been used according to their wavelength: carbon dioxide (CO_2_), semiconductor diode, neodymium-doped yttrium-aluminum-garnet (Nd:YAG), and erbium-doped yttrium-aluminum-garnet (Er:YAG). They are considered to be a less invasive deepithelialization alternative to traditional surgical procedures that present several risks such as pain, edema, and infection [5].

The diode laser is a solid-state semiconductor laser that is emitted in continuous-wave and gated-pulsed modes [5]. Thanks to its different components, it converts electrical energy into light energy, which in turn is converted into heat [6, 7]. Dental laser energy has an affinity for hemoglobin and melanin. It is characterized by wavelengths of 800-980 nm which target especially soft tissues. It is therefore ideal for gum depigmentation [1, 5, 6]. A generally higher absorption with less tissue penetration has been observed for the diode laser compared to the Nd:YAG laser, resulting in deeper coagulation and more severe damage at the tissue surface [8].

According to the results found by Agha and Polenik [9], melanin shows a strong absorption of the diode wavelengths compared to the erbium laser. This results in faster peeling of melanin and a shorter treatment procedure with the diode [10].

**Figure 5:**
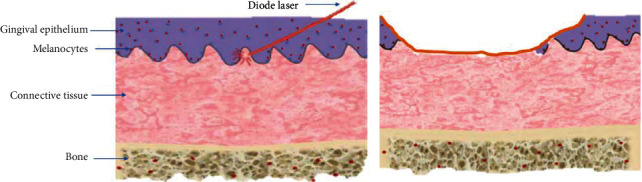
Gingival stripping of the basal layer of the epithelium.

For a proper depigmentation, the basal and suprabasal layers of the gingival epithelium, where the melanocytes are located, must be removed [11]. The use of a laser has the advantage of homogeneously removing the epithelium as well as the rete pegs (Figure 5).

Lasers in soft tissue surgery are associated with a significant reduction of bleeding. According to El Shenawy et al. [12], bleeding was observed in a few patients after conventional surgery, whereas it was absent after laser depigmentation.

Laser depigmentation offers a better visibility, allowing the dental surgeon to act faster and more accurately with sterilization of the wound site [13]. Indeed, Schoop et al. [14] have shown that locally sterile conditions can be achieved thanks to the bactericidal effect of the diode laser.

Khakar et al. [12, 15] found that pain was less in patients treated with laser compared to patients treated with surgery and electrosurgery, probably due to the ability of the laser to seal blood vessels and nerve endings.

These benefits were observed in our case as the patient did not report any infection, swelling, or other complications.

Nevertheless, the use of the laser has some disadvantages such as the fact that it can cause ulcerations and recessions (especially in cases of a thin periodontium), that it remains a relatively expensive tool, resulting in additional costs for the patient [16], and that it presents a significant risk of pigmentation recurrence. Indeed, the recurrence of colorations is the major problem that can be encountered after depigmentation treatments. Several hypotheses have been put forward: the nature of the technique used, the race of the patient [5], and the recolonisation of the treated surfaces with active melanocytes from adjacent tissues [17]. Repigmentation can also be attributed to residual melanocytes left during the operation; they can start to synthesize melanin once activated as suggested by Ginwalla et al. [18]. Nakamura et al. [19] described no repigmentation during the first year following CO_2_ laser depigmentation in 10 patients, but repigmentation was observed in four patients at 24 months. According to Agha and Polenik [9] and Hegde et al. [20], the recurrence rate is lower in the diode laser group compared to those treated with erbium. This can be explained by the fact that there is less chance of finding any unpeeled pigmented cells or particles with the diodes as they penetrate deeper into the tissue.

The patient's ethnicity and tobacco consumption are two factors mentioned in the literature that could promote pigmentation recurrence. Esen et al. [21] observed a recurrence in 2 out of 10 patients, both smokers, in whom they performed gum depigmentation. Smoking cessation may be mandatory to avoid repigmentation. In order to prolong the stability of the results obtained, reducing the impact of other factors like some medications and some habits is necessary [3].

In our case, the use of an 808 nm diode laser for gum depigmentation resulted in a complete healing by the 16th week.

It has also been shown to be a safe and effective method for an acceptable aesthetic result and maximum patient comfort. A longer patient follow-up is required to monitor the onset of repigmentation.

## 4. Conclusion

A diode laser today seems to be an effective and safe technique for melanin depigmentation and holds an undeniable place in the arsenal therapeutic in soft tissue surgery. This technology reduces the level of anxiety and stress in patients with better postoperative outcomes and brings real comfort to the practitioner. However, it is necessary to overcome the skepticism of some practitioners facing this relatively new technology in order to make it an everyday tool to make successful treatments.

## References

[1] Kaya G. Ş., Yavuz G. Y., Sümbüllü M. A., Dayı E. (2012). A comparison of diode laser and Er:YAG lasers in the treatment of gingival melanin pigmentation. *Oral and Maxillofacial Surgery*.

[2] Pasupuleti M. K., Ravindra Reddy N., Roopa D., Sahitya S., Swamy N. (2012). Aesthetic gingival depigmentation procedures: clinical and patient responses. *International Journal of Stomatology & Occlusion Medicine*.

[3] Monteiro L. S., Costa J. A., da Câmara M. I. (2015). Aesthetic depigmentation of gingival smoker’s melanosis using carbon dioxide lasers. *Case Reports in Dentistry*.

[4] Kumar S., Bhat G. S., Bhat K. M. (2012). Development in techniques for gingival depigmentation - An update. *Indian Journal of Dentistry*.

[5] Gupta G. (2011). Management of gingival hyperpigmentation by semiconductor diode laser. *Journal of Cutaneous and Aesthetic Surgery*.

[6] Aoki A., Sasaki K. M., Watanabe H., Ishikawa I. (2004). Lasers in non surgical periodontal therapy. *Periodontology 2000*.

[7] Nakamura Y., Funato A., Wakabayashi H., Matsumoto K. (1992). A study on the removal of the melanin pigmentation of dog gingiva by CO2 laser irradiation. *Journal of Clinical Laser Medicine & Surgery*.

[8] Rastegar S., Jacques S. L., Motamedi M., Kim B. M. (1992). Theoretical analysis of equivalency of high-power diode laser and Nd:YAG laser for coagulation. *Laser-Tissue Interaction III*.

[9] Agha M. T., Polenik P. (2020). Laser Treatment for Melanin Gingival Pigmentations: A Comparison Study for 3 Laser Wavelengths 2780, 940, and 445 nm. *International Journal of Dentistry*.

[10] Patil U. A., Dhami L. D. (2008). Overview of lasers. *Indian Journal of Plastic Surgery*.

[11] Elemek E. (2019). Gingival melanin depigmentation by 810 nm diode laser. *European Journal of Dentistry*.

[12] El Shenawy H. M., Nasry S. A., Zaky A. A., Quriba M. A. (2015). Treatment of gingival hyperpigmentation by diode laser for esthetical purposes. *Macedonian Journal of Medical Sciences*.

[13] Ozbayrak S., Dumlu A., Ercalik-Yalcinkaya S. (2000). Treatment of melanin-pigmented gingiva and oral mucosa by CO_2_ laser. *Oral Surgery, Oral Medicine, Oral Pathology, Oral Radiology, and Endodontology*.

[14] Schoop U., Kluger W., Dervisbegovic S. (2006). Innovative wavelengths in endodontic treatment. *Lasers in Surgery and Medicine*.

[15] Khakar M., Kapoor R., Jayakumar O., Padmalatha S. S., Varghese M. S. (2011). Advantages of 980 nm diode laser treatment in the management of gingival pigmentation. *Journal of Laser Dentistry*.

[16] Lagdive S., Doshi Y., Marawar P. P. (2009). Management of gingival hyperpigmentation using surgical blade and diode laser therapy: a comparative study. *Journal of Oral Laser Applications*.

[17] Perlmutter S., Tal H. (1986). Repigmentation of the gingiva following surgical injury. *Journal of Periodontology*.

[18] Ginwalla T. M., Gomes B. C., Varma B. R. (1966). Surgical removal of gingival pigmentation. (a preliminary study). *Journal of the Indian Dental Association*.

[19] Nakamura Y., Hossain M., Hirayama K., Matsumoto K. (1999). A clinical study on the removal of gingival melanin pigmentation with the CO2 laser. *Lasers in Surgery and Medicine*.

[20] Hegde R., Padhye A., Sumanth S., Jain A. S., Thukral N. (2013). Comparison of surgical stripping; erbium-doped:yttrium, aluminum, and garnet laser; and carbon dioxide laser techniques for gingival depigmentation: a clinical and histologic study. *Journal of Periodontology*.

[21] Esen E., Haytac M. C., Oz I. A., Erdoğan O., Karsli E. D. (2004). Gingival melanin pigmentation and its treatment with the CO_2_ laser. *Oral Surgery, Oral Medicine, Oral Pathology, Oral Radiology and Endodontology*.

